# Impact of frontal white matter hyperintensity on instrumental activities of daily living in elderly women with Alzheimer disease and amnestic mild cognitive impairment

**DOI:** 10.1371/journal.pone.0172484

**Published:** 2017-03-02

**Authors:** Noriko Ogama, Takashi Sakurai, Toshiharu Nakai, Shumpei Niida, Naoki Saji, Kenji Toba, Hiroyuki Umegaki, Masafumi Kuzuya

**Affiliations:** 1 Center for Comprehensive Care and Research on Memory Disorders, National Center for Geriatrics and Gerontology, Obu, Japan; 2 Department of Community Healthcare and Geriatrics, Nagoya University Graduate School of Medicine, Nagoya, Japan; 3 NeuroImaging and Informatics, National Center for Geriatrics and Gerontology, Obu, Japan; 4 Medical Genome Center, National Center for Geriatrics and Gerontology, Obu, Japan; Nathan S Kline Institute, UNITED STATES

## Abstract

**Background:**

Instrumental activities of daily living (IADL) start to decline during the progression of amnestic mild cognitive impairment (aMCI) to Alzheimer disease (AD). Cognitive and physical decline are involved in the loss of functional independence. However, little is known about AD-related neural change that leads to IADL impairment. The purpose of this study was to clarify the effects of regional white matter hyperintensity (WMH) on IADL impairment in persons with AD and aMCI.

**Methods:**

The participants were 347 female subjects aged 65–85 years diagnosed with AD (n = 227), aMCI (n = 44) or normal cognition (n = 76). IADL was assessed by the Lawton Index. Cognition, mood and mobility function were evaluated by comprehensive geriatric assessment batteries. WMH and brain atrophy were analyzed with brain magnetic resonance imaging, using an automatic segmentation program. Regional WMH was measured in the frontal, temporal, occipital and parietal lobes.

**Results:**

Ability to carry out IADL of shopping, food preparation, mode of transportation, responsibility for own medication, and ability to handle finances was obviously impaired in the early stage of AD. Frontal WMH was specifically associated with disability to do shopping and food preparation even after adjusting for several confounders including brain atrophy.

**Conclusions:**

IADL subcategories were differentially impaired along with cognitive status in persons with AD and aMCI. Frontal WMH was an important predictor of impaired ability to do shopping and food preparation. A preventive strategy for WMH might lead to suppression of IADL disability and slow the progression of AD.

## Introduction

White matter hyperintensity (WMH) is observed as hyperintense areas in T2-weighted and fluid-attenuated inversion recovery (FLAIR) images, and isointense or hypointense areas in T1-weighted images on magnetic resonance (MR) images. WMH is frequently found in the aging brain, and occurs in both the periventricular and deep subcortical areas. Previous studies showed that WMH manifests as several geriatric syndromes [1], including cognitive decline [2, 3], motor disturbance [4], urinary incontinence [5] and depressive symptoms [6], when WMH is sufficiently diffuse and confluent [7].

Instrumental activities of daily living (IADL) are composed of several activities required to maintain independent living such as shopping, cooking, handling medication and managing finances. These activities require more complex behaviors and higher executive function than does self-maintenance of basic ADL. Therefore, IADL start to decline during the progression of amnestic mild cognitive impairment (aMCI) to Alzheimer disease (AD) [8]. Impairment of daily life functions worsens with the clinical stage of AD, and increases caregivers’ burden [9]. It is crucial to elucidate the mechanism underlying the decline in IADL in persons with AD. The following have been reported to be associated with IADL decline; older age [10], female sex [10], lower education [10], cognitive decline [11–14], executive dysfunction [11, 13–15], depressive symptoms [16–19] and physical dysfunction [20–23]. Recently, a relationship between WMH and IADL decline has been suggested [24–29]. However, the precise roles of WMH in IADL decline remain uncertain in persons with dementia, because other factors such as brain atrophy may potentially modulate functional independence.

The aim of this study was to identify the specific interaction between regional WMH and impairment of IADL subdomains in persons with AD and aMCI. The goals of this study were: (1) to identify impaired IADL items in several cognitive stages, and (2) to determine the regional and volume effects of WMH on IADL abilities after adjusting for possible confounders and brain atrophy.

## Materials and methods

### Participants

This study was approved by the Ethical Review Board of Japan’s National Center for Geriatrics and Gerontology (NCGG). Written informed consent was obtained from candidate subjects and their caregivers prior to participation in the study. We enrolled 347 outpatients consecutively at their initial visit to our hospital in the period from 2010 to 2013. We selected only female subjects, because housekeeping activities and life style differ by gender. The subjects were aged 65–85 years, with Mini Mental State Examination (MMSE) ≧ 15, Barthel Index ≧ 80, and a diagnosis of AD (n = 227), aMCI (n = 44) or normal cognition (NC, n = 76). Diagnoses of AD and aMCI were based on the National Institute on Aging/Alzheimer's Association workgroups on diagnostic guidelines for Alzheimer disease [30, 31]. AD patients were subclassified into three groups by their total MMSE scores: AD 30–24 (MMSE score range 30–24; n = 46), AD 23–20 (23–20; n = 85) and AD 19–15 (19–15; n = 96). Persons with NC attended the NCGG hospital with suspected memory disorder, but were assessed as having normal cognition. Patients with a history of stroke or cortical lesions on MR images, severe conditions such as cardiac failure, renal disorder, or liver dysfunction, or neurological disorders other than AD were excluded from this study.

### Evaluation of functional status, cognition, mood and physical function

Clinical data were obtained from the NCGG Biobank, which collects and stores biological material and associated clinical information for biomedical research. All the participants underwent assessment with comprehensive geriatric assessment batteries. The patients’ basic ADL and IADL were assessed by the Barthel Index and the Lawton Index, respectively [32, 33]. The Lawton Index is composed of eight items (telephone use, shopping, food preparation, housekeeping, laundry, mode of transportation, responsibility for own medication, and ability to handle finances). Global cognitive function was tested using MMSE and Alzheimer's Disease Assessment Scale (ADAS). Memory impairment was assessed by the Wechsler Memory Scale-Revised (WMS-R) logical memory I and II. Frontal lobe function was evaluated by the Frontal Assessment Battery (FAB) [34]. Attention was evaluated by the digit span forward and backward. Mood disturbance was assessed by the self-rated Geriatric Depression Scale 15 items and Vitality Index. Mobility function was assessed using the Fall Risk Index (FRI), Timed Up and Go test (TUG), and grip strength. FRI is composed of 21 questions to measure physical function, geriatric syndrome, and environmental hazards [35].

### Evaluation of WMH and brain atrophy

MR images were obtained using 1.5T MR scanners (Siemens Avanto, Germany; or Philips Ingenia, Netherlands) with T1-weighted, T2-weighted and FLAIR images [5]. WMH, intracranial (IC), parenchyma (PAR), cerebrospinal fluid (CSF) and ventricle (VCL) volumes were measured by using an automatic segmentation application (SNIPER, Software for Neuro-Image Processing in Experimental Research: Department of Radiology, Leiden University Medical Center, Netherlands). Details of the MR imaging protocol and SNIPER are described elsewhere [5, 36]. Briefly, WMH was automatically segmented in each brain lobe (frontal, temporal, occipital and parietal lobes) and divided into periventricular hyperintensity (PVH) or deep white matter hyperintensity (DWMH). IC volume included brain parenchyma (sum of total gray and white matter including WMH) and CSF. Global brain atrophy was assessed by PAR which is the subtraction of CSF from IC. WMH, PAR, CSF and VCL were divided by the IC volume to minimize bias from each patient’s brain size.

### Statistical analysis

**Fig 1:**
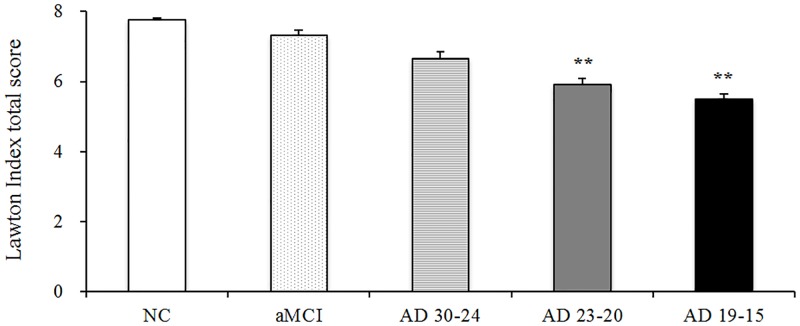
Difference of Lawton Index total score in each clinical stage of AD, aMCI and NC. The patients were divided into five groups: normal cognition (NC), amnestic mild cognitive impairment (aMCI) and Alzheimer disease (AD). AD patients were subclassified into three subgroups by Mini-Mental State Examination score: AD 30–24, AD 23–20 and AD 19–15 (MMSE score range 30–24, 23–20 and 19–15). Differences between groups were analyzed by analysis of covariance (Bonferroni) with adjustment for age. Lawton Index total score was significantly lowered in persons with cognitive impairment. ** p < 0.01, * p < 0.05 compared with NC.

**Fig 2:**
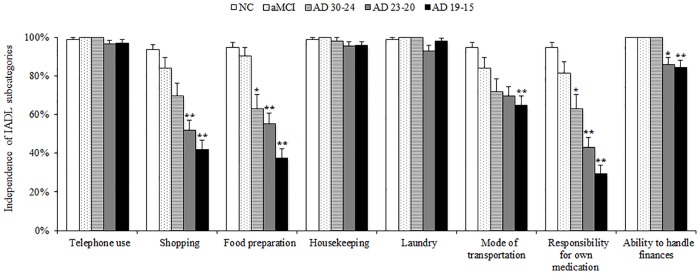
Difference of Lawton Index subcategories in each clinical stage of AD, aMCI and NC. Differences between groups were analyzed by analysis of covariance (Bonferroni) with adjustment for age. IADL subdomains such as shopping, food preparation, mode of transportation, responsibility for own medication and ability to handle finances were significantly lowered with cognitive impairment. ** p < 0.01, * p < 0.05 compared with NC.

All analyses were performed using the Japanese version of SPSS for Windows version 22.0 (IBM Corporation, Armonk, NY, USA). Differences in clinical profiles between AD, aMCI and NC were examined using the Kruskal-Wallis test (Table 1). To assess the differences in IADL among NC, aMCI and the three AD subgroups (Figs 1 and 2), we performed an analysis of covariance controlling for age, with Lawton Index as a dependent variable and each cognitive groups as an independent variable. When assessing the relationship between WMH and cognitive function, we performed multiple regression analysis controlled for age and education, in which dependent variables were each cognitive parameter, while independent variable was total WMH.

**Table 1:** Clinical characteristics of study participants (N = 347).

	AD (n = 227)	aMCI (n = 44)	NC (n = 76)
Age, years	77.8 (4.9) *^,^^†^	75.1 (5.6)	73.1 (4.7)
Education, years	9.9 (2.0) *^,^^†^	10.8 (2.0)	11.3 (2.3)
Barthel Index	98.3 (4.1) *	99.7 (1.3)	99.5 (1.9)
Lawton Index	5.9 (1.7) *^,^^†^	7.3 (1.1)	7.7 (0.7)
Telephone use	0.97 (0.16)	1.00 (0.00)	0.99 (0.11)
Shopping	0.51 (0.50) *^,^^†^	0.84 (0.37)	0.93 (0.25)
Food preparation	0.49 (0.50) *^,^^†^	0.90 (0.30)	0.95 (0.22)
Housekeeping	0.96 (0.20)	1.00 (0.00)	0.99 (0.11)
Laundry	0.96 (0.18)	1.00 (0.00)	0.99 (0.11)
Mode of transportation	0.68 (0.47) *	0.84 (0.37)	0.95 (0.22)
Responsibility for own medication	0.41 (0.49) *^,^^†^	0.81 (0.39)	0.95 (0.22)
Ability to handle finances	0.88 (0.32) *^,^^†^	1.00 (0.00)	1.00 (0.00)
**Cognitive function**			
Mini-Mental State Examination	20.4 (3.5) *^,^^†^	24.8 (2.6) *	28.4 (1.9)
Alzheimer's Disease Assessment Scale	17.3 (5.4) *^,^^†^	10.3 (3.3) *	5.3 (2.3)
WMS-R logical memory I	3.4 (3.3) *^,^^†^	6.5 (4.5) *	17.8 (5.0)
WMS-R logical memory II	0.5 (1.1) *^,^^†^	1.7 (3.2) *	12.6 (5.9)
Frontal Assessment Battery	9.5 (2.6) *^,^^†^	11.5 (2.8) *	13.2 (2.5)
Digit span forwards	5.1 (1.0) *	5.5 (1.0)	5.7 (1.1)
Digit span backwards	3.2 (0.9) *	3.6 (0.8)	4.0 (1.1)
**Mood disturbance**			
Geriatric Depression Scale	4.4 (2.6)	3.8 (2.5)	4.6 (3.1)
Vitality Index	9.0 (1.1) *^,^^†^	9.8 (0.4)	9.7 (0.6)
**Mobility function**			
Fall Risk Index	5.6 (3.8) ^†^	3.9 (3.3)	4.6 (3.6)
Timed Up and Go, sec	11.2 (3.8) *^,^^†^	10.1 (3.6)	9.4 (3.0)
Grip strength, kg	17.6 (5.5) *^,^^†^	20.9 (3.7)	20.8 (5.2)
**MR imaging**			
IC, mL	1322.6 (95.5) *	1350.5 (104.4)	1368.0 (101.6)
WMH, mL, % of IC	18.0 (17.9) *^,^^†^, 1.36%	13.1 (16.6), 0.97%	7.3 (8.4), 0.54%
Frontal lobe, mL, % of IC	10.2 (9.8) *^,^^†^, 0.77%	6.9 (7.7), 0.52%	4.3 (4.4), 0.32%
Temporal lobe, mL, % of IC	1.2 (1.4) *^,^^†^, 0.09%	1.1 (2.3), 0.08%	0.4 (0.6), 0.03%
Occipital lobe, mL, % of IC	0.5 (0.8) *, 0.04%	0.5 (0.7), 0.04%	0.3 (0.5), 0.02%
Parietal lobe, mL, % of IC	6.1 (7.1) *^,^^†^, 0.46%	4.6 (6.9), 0.34%	2.2 (4.0), 0.17%
Periventricular area, mL, % of IC	17.0 (17.5) *^,^^†^, 1.28%	12.1 (15.9), 0.90%	6.6 (8.0), 0.49%
Deep subcortical areas, mL, % of IC	1.1 (1.4) *, 0.08%	1.0 (1.4), 0.08%	0.7 (1.0), 0.05%
PAR, mL, % of IC	989.4 (78.3) *^,^^†^, 74.8%	1035.3 (87.1), 76.7%	1044.4 (83.8), 76.4%
CSF, mL, % of IC	333.7 (47.3) *^,^^†^, 25.2%	315.6 (47.2), 23.4%	323.5 (50.1), 23.6%
VCL, mL, % of IC	57.3 (19.3) *^,^^†^, 4.32%	47.5 (14.9), 3.50%	50.6 (20.9), 3.69%

Data are presented as mean (standard deviation). Patients were divided into three groups: Alzheimer disease (AD), amnestic mild cognitive impairment (aMCI) and normal cognition (NC). Data of MR imaging for NC was from 68 participants.

*p < 0.05, versus NC.

^†^p < 0.05, versus aMCI (Kruskal-Wallis test).

Abbreviations: AD, Alzheimer disease; aMCI, amnestic mild cognitive impairment; CSF, cerebrospinal fluid; IC, intracranial; MR, magnetic resonance; NC, normal cognition; PAR, parenchyma; VCL, ventricular; WMH, white matter hyperintensity; WMS-R, Wechsler Memory Scale-Revised.

To explore potential risk factors for IADL impairment, we used single regression and multiple regression analyses. First, we performed single regression analysis (Table 2). We calculated the coefficient of determination of each clinical index to identify the relationship with total IADL. Dependent variable was Lawton Index total score, and independent variables were age, education and clinical parameters of cognitive function, mood, and mobility function. Second, we conducted multiple regression analyses to examine the relationship between various MR imaging parameters and total IADL (Table 3). Independent variables were age, total WMH, PAR and VCL in model 1, and age, regional WMH, PAR and VCL in model 2.

**Table 2:** Association of clinical characteristics and IADL in AD and aMCI.

	*β*	(95% CI)	R^2^
Age	-0.29	(-0.13; -0.06)	0.08
Education	0.10	(-0.01; 0.19)	0.01
**Cognitive function**			
MMSE	0.36	(0.11; 0.21)	0.13
ADAS	-0.42	(-0.16; -0.09)	0.18
WMS-R logical memory I	0.20	(0.04; 0.15)	0.04
WMS-R logical memory II	0.12	(-0.01; 0.24)	0.01
FAB	0.26	(0.09; 0.24)	0.07
Digit span forwards	0.08	(-0.08; 0.36)	0.01
Digit span backwards	0.07	(-0.11; 0.38)	0.01
**Mood disturbance**			
GDS	-0.17	(-0.19; -0.03)	0.03
Vitality Index	0.43	(0.49; 0.82)	0.18
**Mobility function**			
FRI	-0.23	(-0.16; -0.05)	0.05
TUG	-0.20	(-0.14; -0.04)	0.04
Grip strength	0.27	(0.04; 0.14)	0.07

Single regression analysis. The dependent variable was the Lawton Index total score.

Independent variables were age, education and each clinical index. R^2^ is the proportion of explained variance of the Lawton Index total score. Abbreviations: AD, Alzheimer Disease; ADAS, Alzheimer's Disease Assessment Scale; aMCI, amnestic mild cognitive impairment; CI, confidence interval; FAB, Frontal Assessment Battery; FRI, Fall Risk Index; GDS, Geriatric Depression Scale; IADL, Instrumental activities of daily living; MMSE, Mini-Mental State Examination; TUG, Timed Up and Go; WMS-R, Wechsler Memory Scale-Revised.

**Table 3:** Association of WMH and brain atrophy with IADL in AD and aMCI.

	*β*	(95% CI)	p value
Model 1			
Age	-0.19	(-0.11; -0.02)	0.004
Total WMH	-0.20	(-0.41; -0.10)	0.001
PAR	0.17	(0.03; 0.17)	0.008
VCL	-0.01	(-0.17; 0.14)	0.893
Model 2			
Age	-0.19	(-0.10; -0.02)	0.004
WMH in frontal lobe	-0.33	(-1.33; -0.24)	0.005
WMH in temporal lobe	0.03	(-2.85; 3.69)	0.802
WMH in occipital lobe	-0.08	(-7.46; 2.92)	0.390
WMH in parietal lobe	0.15	(-0.46; 1.42)	0.313
PAR	0.16	(0.02; 0.16)	0.013
VCL	0.00	(-0.15; 0.16)	0.989

Multiple regression analysis with forced entry method. The dependent variable was the Lawton Index total score. Age, total WMH, PAR and VCL were entered as independent variables in model 1, and age, regional WMH, PAR and VCL were entered as independent variables in model 2. Abbreviations: AD, Alzheimer disease; aMCI, amnestic mild cognitive impairment; CI, confidence interval; IADL, Instrumental activities of daily living; PAR, parenchyma; VCL, ventricular; WMH, white matter hyperintensity.

Finally, to identify independent predictors of IADL impairment, we created two sets of multiple regression analysis. Dependent variable was Lawton Index total score and Lawton Index subcategories in Tables 4 and 5, respectively. The MR imaging parameters and clinical indices showing the highest coefficient of determination in Table 2 were entered as independent variables (Model 1). MR imaging parameters, clinical indices (p < 0.1 in previous model) and classical confounders known to influence IADL impairment (i.e., global cognitive function, depressive mood and gait performance [12, 16–19, 20–23]) were entered as independent variables (Model 2). Statistical significance was defined as p < 0.05.

**Table 4:** Independent risk factors for IADL impairment in AD and aMCI.

Model 1	*β*	(95% CI)	p value
Age	0.05	(-0.03; 0.06)	0.535
WMH in frontal lobe	-0.20	(-0.84; -0.16)	0.004
PAR	0.15	(0.01; 0.16)	0.036
ADAS	-0.15	(-0.09; 0.00)	0.049
Vitality Index	0.36	(0.33; 0.76)	< 0.001
Grip strength	0.21	(0.03; 0.12)	0.002
Model 2			
Age	0.06	(-0.03; 0.07)	0.415
WMH in frontal lobe	-0.19	(-0.82; -0.14)	0.005
PAR	0.16	(0.01; 0.16)	0.020
ADAS	-0.12	(-0.09; 0.02)	0.171
Vitality Index	0.33	(0.29; 0.72)	< 0.001
Grip strength	0.19	(0.02; 0.12)	0.010
MMSE	0.03	(-0.06; 0.09)	0.720
GDS	-0.19	(-0.20; -0.04)	0.003
TUG	0.01	(-0.05; 0.06)	0.885

Multiple regression analysis with forced entry method. The dependent variable was the Lawton Index total score. Model 1: Age, frontal WMH, PAR and clinical indices (ADAS, Vitality Index and grip strength, which exhibited the highest R^2^ values in each clinical section in Table 2) were entered as independent variables. Model 2: Age, frontal WMH, PAR, clinical indices (p < 0.1 in model 1) and classical confounders (MMSE, GDS and TUG) were entered as independent variables. Abbreviations: AD, Alzheimer disease; aMCI, amnestic mild cognitive impairment; CI, confidence interval; GDS, Geriatric Depression Scale; IADL, Instrumental activities of daily living; MMSE, Mini-Mental State Examination; PAR, parenchyma; TUG, Timed Up and Go; WMH, white matter hyperintensity.

**Table 5:** Independent risk factors for impairment of IADL subcategories in AD and aMCI.

	Shopping			Food preparation			Mode of transportation			Responsibility for own medication			Ability to handle finances		
Model 1	OR	(95% CI)	p value	OR	(95% CI)	p value	OR	(95% CI)	p value	OR	(95% CI)	p value	OR	(95% CI)	p value
Age	0.99	(0.91; 1.08)	0.819	1.07	(0.98; 1.18)	0.136	0.94	(0.86; 1.02)	0.155	1.00	(0.93; 1.09)	0.971	1.10	(0.96; 1.25)	0.171
WMH in frontal lobe	0.44	(0.23; 0.85)	0.014	0.42	(0.21; 0.83)	0.012	0.82	(0.48; 1.42)	0.481	0.68	(0.38; 1.22)	0.198	0.37	(0.17; 0.78)	0.009
PAR	1.09	(0.96; 1.25)	0.187	1.15	(1.00; 1.32)	0.056	1.09	(0.95; 1.24)	0.221	1.03	(0.91; 1.17)	0.642	1.03	(0.86; 1.23)	0.785
ADAS	0.95	(0.88; 1.03)	0.223	0.92	(0.85; 1.00)	0.057	0.94	(0.87; 1.01)	0.099	0.94	(0.87; 1.02)	0.115	0.97	(0.86; 1.09)	0.579
Vitality Index	2.33	(1.53; 3.53)	< 0.001	2.15	(1.41; 3.29)	< 0.001	1.03	(0.72; 1.47)	0.869	2.27	(1.51; 3.42)	< 0.001	1.57	(0.93; 2.63)	0.090
Grip strength	1.15	(1.06; 1.26)	0.001	1.21	(1.10; 1.33)	< 0.001	1.05	(0.97; 1.14)	0.199	1.05	(0.97; 1.14)	0.236	0.99	(0.88; 1.12)	0.899
Model 2															
Age	0.98	(0.90; 1.07)	0.618	1.10	(0.99; 1.21)	0.074	0.92	(0.86; 0.99)	0.017	0.94	(0.89; 1.00)	0.064	0.98	(0.89; 1.08)	0.642
WMH in frontal lobe	0.43	(0.23; 0.80)	0.008	0.43	(0.21; 0.86)	0.017	0.86	(0.56; 1.31)	0.477	0.82	(0.54; 1.24)	0.346	0.64	(0.38; 1.07)	0.086
PAR	1.11	(0.98; 1.26)	0.099	1.16	(1.01; 1.34)	0.042	1.07	(0.96; 1.20)	0.213	1.03	(0.93; 1.15)	0.533	1.00	(0.87; 1.15)	0.982
ADAS	N/A			0.91	(0.82; 1.00)	0.053	0.91	(0.85; 0.98)	0.010	N/A			N/A		
Vitality Index	2.02	(1.38; 2.95)	< 0.001	2.18	(1.40; 3.39)	0.001	N/A			2.12	(1.54; 2.92)	< 0.001	1.39	(0.98; 1.97)	0.066
Grip strength	1.07	(0.99; 1.16)	0.104	1.21	(1.09; 1.34)	< 0.001	N/A			N/A			N/A		
MMSE	1.13	(1.01; 1.26)	0.034	0.95	(0.82; 1.09)	0.447	0.93	(0.84; 1.03)	0.138	1.18	(1.09; 1.28)	< 0.001	1.21	(1.05; 1.38)	0.008
GDS	0.92	(0.81; 1.05)	0.225	0.93	(0.80; 1.07)	0.293	0.87	(0.77; 0.97)	0.012	0.90	(0.81; 1.00)	0.058	0.86	(0.74; 1.00)	0.056
TUG	0.96	(0.84; 1.09)	0.512	0.95	(0.82; 1.11)	0.551	0.91	(0.83; 0.99)	0.028	1.03	(0.95; 1.12)	0.451	1.07	(0.95; 1.20)	0.302

Multiple logistic regression analysis with forced entry method. The dependent variables were each of the Lawton Index subcategories. Model 1: Age, frontal WMH, PAR and clinical indices (ADAS, Vitality Index and grip strength, which exhibited the highest R^2^ values in each clinical section in Table 2) were entered as independent variables. Model 2: Age, frontal WMH, PAR, clinical indices (p < 0.1 in model 1) and classical confounders (MMSE, GDS and TUG) were entered as independent variables. N/A = model was not run because coefficients in the previous model were p > 0.1. Abbreviations: AD, Alzheimer Disease; ADAS, Alzheimer's Disease Assessment Scale; aMCI, amnestic mild cognitive impairment; CI, confidence interval; GDS, Geriatric Depression Scale; IADL, instrumental activities of daily living; MMSE, Mini-Mental State Examination; PAR, parenchyma; TUG, Timed Up and Go; WMH, white matter hyperintensity.

## Results

### Clinical data

Clinical profiles of the study participants are shown in Table 1. The mean (± SD) age of the AD, aMCI and NC groups was 77.8 ± 4.9, 75.1 ± 5.6 and 73.1 ± 4.7 years, respectively. Total score of the Lawton Index was markedly lowered (indicating worsening) in AD, and slightly lowered in aMCI. Basic ADL were impaired only in the AD group. Performance in all cognitive tests was significantly impaired in AD, and that in MMSE, ADAS, WMS-R logical memory and FAB was worse in aMCI. Vitality and mobility function were also impaired in persons with AD.

In MR imaging analysis, mean IC volume in AD, aMCI and NC were 1322.6 ± 95.5 mL, 1350.5 ± 104.4 mL and 1368.0 ± 101.6 mL, and mean WMH volume was 18.0 ± 17.9 mL, 13.1 ± 16.6 mL and 7.3 ± 8.4 mL respectively. AD patients exhibited significantly greater WMH compared with aMCI and NC, particularly in the frontal, temporal and parietal lobes, as well as more severe brain atrophy. Moreover, aMCI tended to have slightly greater WMH than NC but this difference was not statistically significant.

### Differences of IADL impairment with cognitive status

To assess the difference in IADL subdomains with various cognitive status, AD subjects were classified into three subgroups by total MMSE score; AD 30–24, AD 23–20 and AD 19–15. Lower Lawton Index total score was observed in patients with worse MMSE performance (Fig 1). Ability to do shopping, food preparation, mode of transportation, responsibility for own medication, and ability to handle finances were significantly impaired in subjects with lower MMSE score, while ability to use a telephone, do housekeeping, and laundry were maintained even in the AD 19–15 group (Fig 2). In the aMCI group, there was no significant impairment in IADL subdomains. However, ability to do shopping, food preparation, mode of transportation and responsibility for own medication showed a tendency to be worse.

### Association of WMH and cognitive function

Association between total WMH and cognitive function was tested using multiple regression analysis controlled for age and education. The results indicated that WMH was significantly associated with MMSE (β = –0.15, p = 0.016), ADAS (β = 0.18, p = 0.003) and FAB (β = –0.16, p = 0.019), but not with WMS-R logical memory I (β = –0.13, p = 0.055), logical memory II (β = 0.00, p = 1.00), digit span forward (β = –0.07, p = 0.260) and digit span backward (β = –0.12, p = 0.077).

### Association of clinical characteristics and MR imaging parameters with IADL

Clinical factors influencing IADL were tested using single regression (Table 2). To estimate predictors of the Lawton Index total score, we calculated the coefficients of determination. The results revealed that ADAS, Vitality Index and grip strength were the strongest predictors of the Lawton Index total score in cognition, mood, and mobility.

The effects of WMH and brain atrophy on Lawton Index total score were tested using multiple regression (Table 3). Because PAR and CSF were strongly correlated (r = –1.00, p < 0.001), CSF was not included as an independent variable. In model 1, age, total WMH, PAR and VCL were entered as independent variables. The results revealed that age, total WMH and PAR were independently associated with the Lawton Index total score. In the model 2, age, regional WMH, PAR and VCL were entered as independent variables. Based on this analysis, age, WMH in the frontal lobe and PAR were extracted as predictors of IADL impairment.

### Independent risk factors for IADL impairment (Lawton Index total score)

We explored independent risks for IADL impairment in two models of multiple regression with a forced entry method (Table 4). Model 1 revealed that WMH in frontal lobe, PAR, ADAS, Vitality Index and grip strength were independently associated with Lawton Index total score. In Model 2, in addition to Vitality Index, grip strength and GDS, WMH in frontal lobe and PAR were also independently predictor for IADL impairment.

### Independent risk factors for impairment of IADL subcategories

Finally, we conducted multiple logistic regression analyses to explore independent risk factors for impairment on IADL subcategories (Table 5). In model 1, WMH in the frontal lobe, Vitality Index and grip strength were independent risk factors for impaired ability to do shopping and food preparation. In addition, WMH in the frontal lobe was significantly associated with ability to handle finance. Global cognition and brain atrophy showed a tendency to be associated with disability of food preparation.

In model 2, WMH in the frontal lobe, Vitality Index and MMSE were independent risk factors for impairment of ability to do shopping. For impairment of food preparation, WMH in the frontal lobe, brain atrophy, Vitality Index and grip strength were predictive factors. However, for mode of transportation, responsibility for own medication and ability to handle finances, neither regional WMH nor brain atrophy were extracted as risk factors. Age, global cognition, mood and mobility function were extracted as independent risk factors for impairment of the three IADL subcategories.

## Discussion

The present study revealed two major findings. Firstly, IADL subcategories were differentially impaired in several cognitive status: ability to do shopping, food preparation, mode of transportation, responsibility for own medication and ability to handle finances were readily impaired in the early stage of AD, whereas telephone use, housekeeping, and laundry were spared even in AD 19–15. Use of these sustained IADL activities could be beneficial to support independent living in persons with AD. Secondly, WMH was associated with IADL impairment independent of possible confounders such as age, cognitive status, mood disturbance, and mobility impairment. Especially, WMH in the frontal lobe was critical for impaired ability to do shopping and food preparation. Since the diagnosis and severity of dementia are usually determined by the degree of functional decline, a preventive strategy against WMH might help to maintain IADL, resulting in slower disease progression in persons with AD.

Several cross-sectional and longitudinal studies have shown that extensive WMH is related to more marked IADL disturbance [24, 27–29] and a considerable risk of rapid loss of independence in a short period [25, 26]. An association of WMH with impairment of basic ADL and IADL has been demonstrated in a dementia cohort [27]. Regarding the regional effects of WMH on IADL, involvement of periventricular WMH in anterior and middle brain areas has been suggested [29]. The results of this study extended these observations and clearly demonstrated that frontal WMH is an independent risk for IADL impairment, particularly for shopping and food preparation. To carry out activities of shopping and food preparation, executive function for planning, selecting information and shifting behavior appropriately with multiple steps, as well as mobility function may be needed. The current study found relationship between WMH and global cognitive function, and association of global cognition with IADL impairment (Table 2). It seems plausible that WMH impacts on global cognitive function, and has secondary downstream effects on complex IADL at least in part. However, both of frontal WMH and cognitive function were shown to be independent risk for disability of shopping and food preparation (Table 5). WMH might be influencing IADL ability without mediated by cognitive function. However, since this study design is cross-sectional, causal relationship has to be carefully considered and further prospective studies are need. In contrast, WMH was not independently associated with responsibility for own medication and handling finance. Distinct information processing networks or neural activity in different cortical areas may be required for several IADL subcategories.

Mobility function was measured by TUG and grip strength in this study. Grip strength was independently associated with food preparation, while TUG was associated with mode of transportation. Our previous study showed that WMH was not correlated with muscle strength, but was closely associated with TUG [4]. TUG represents total gait function, including standing up from sitting, walking, turning and balance. Slowed gait speed is an important predictor of IADL disability [20–23], and walking ability and grip strength predict impairment of cooking, shopping and housework [21]. Physical frailty is also a strong predictor of functional disability [37, 38]. Interestingly, motor performance did not show an association with responsibility for own medication and handling of finances. These IADL activities may not always require good mobility function.

The frontal lobe is closely associated with executive function [39], which contributes to functional independence in the elderly [11, 13–15]. Vidoni et al. found an association of decreased gray matter volume in the medial frontal and temporal-parietal area with loss of functional independence in early AD [40]. Roy et al. showed that middle frontal and orbitofrontal hypometabolism was associated with IADL impairment, and baseline middle frontal and posterior cingulate hypometabolism was related to greater progression of IADL decline by ^18^F-fluorodeoxyglucose positron emission tomography study [41]. Although we could not determine the changes of regional gray matter volume in this study, indices of global brain atrophy were related to disability of food preparation. A relationship between subcortical hyperintensities in cholinergic pathways and cognitive impairment, particularly frontal dysfunction, has been suggested by several previous studies [42–44]. It seems likely that the mechanism underlying the correlation between frontal WMH and functional dependence is largely attributable to disconnection of major projecting fibers and adjacent associated fibers in the frontal lobe integrating various neural information for complex executive function and behavior [4].

In this context, our study participants had relatively high WMH volume compared with other large studies [45, 46]. The volume of WMH in AD was 18.0 + 17.9 mL, 1.36% of IC, corresponding to grade 2–3 of the Fazekas scale [47, 48]. Most previous studies on WMH concluded that WMH manifests as several syndromes such as cognitive decline and physical disturbance, when WMH is sufficiently diffuse and confluent [7]. However, the existence of asymptomatic patients with extensive WMH has been occasionally reported [7]. A diffusion tensor imaging study revealed that the correlation of deep white matter tract damage and cognitive decline is not linear, and cognitive decline appears when fractional anisotropy is below the specific threshold level despite extensive WMH [49]. Nevertheless, WMH in our study participants showed a close association with IADL impairment, which suggests that IADL activities are sensitive to impairment of frontal brain networks and subcortical structural damage.

WMH is composed of heterogeneous pathologic changes, including axonal and myelin loss and pallor, scattered microinfarcts, astrogliosis, dilatation of perivascular spaces, and cerebral amyloid angiopathy (CAA) [7, 50]. CAA is found in 80–100% of AD patients, and induces cerebral hypoperfusion in the white matter as a result of amyloid β deposition in cortical microvessels [7]. There is accumulating evidence that age-related WMH is most prevalent in the frontal areas of elderly persons, especially those with hypertension [7, 51], which suggests that the pathogenesis of WMH is most likely of ischemic origin as a result of hypertensive small vessel disease, whereas WMH in AD patients shows more posterior involvement [3, 52]. A number of potential risk factors for WMH have been indicated in previous studies, including aging, hypertension, and smoking. Factors associated with the acceleration of the development of WMH include diabetes, dyslipidemia, inflammation, oxidative stress, and sleeping disorders in older adults [53]. Therapeutic interventions to target these risk factors could be valuable for prevention of WMH progression, particularly frontal WMH, to delay the progression of functional loss in elderly persons with AD.

Previous studies demonstrated a correlation between IADL performance and several cognitive domains such as episodic memory, speed of processing and verbal ability [11, 14]. A systematic review revealed that functional status that can specifically be attributed to cognition is modest, while general cognitive function measured by standard screening tests showed a strong correlation with functional status [12]. Our study agreed with this notion, because global cognitive function evaluated by MMSE and ADAS was related to IADL subcategories, whereas specific cognitive tests for memory and attention were not associated with IADL activities (Table 2).

Depressive mood and low vitality were extracted as independent risks for IADL disturbance in the current study. A complex relationship between depressive symptoms and functional decline has been reported [16–19]. Although the precise role of depression in IADL disturbance is not fully understood, elderly with a depressive mood or lower vitality might lose motivation and become reluctant to engage in social interactions, which consequently leads to functional disturbance [9, 16]. Nyunt et al. have shown that an improvement in depressive symptoms can restore IADL ability in community-dwelling older persons [18]. Early diagnosis and therapeutic intervention for mood disturbance may prevent the progression of IADL disturbance in persons with dementia.

This study has several limitations. Firstly, the study design was cross-sectional. Therefore, any causal relationship between WMH and IADL impairment has to be carefully considered. However, the present study contained a large number of subjects with relatively homogenous profiles: women with very mild to moderate cognitive impairment without an obvious deficit in basic ADL. Furthermore, we investigated a wide range of confounding factors for functional independence, which might be pivotal in exploring clinically relevant factors associated with IADL impairment. Secondly, although this study used an automatic segmentation program to analyze WMH volume, we could not distinguish the roles of PVH and DWMH, because the automatic segmentation of WMH mostly recognized PVH, and we analyzed total WMH volume including PVH and DWMH. In this connection, DeCarli et al. suggested that the classification of PVH and DWMH is arbitrary, and causal factors for PVH and DWMH reflect total WMH volume [54].

In conclusion, among IADL subcategories, shopping, food preparation, mode of transportation, responsibility for own medication, and ability to handle finances readily impaired with cognitive impairment in elderly persons with AD. WMH in the frontal lobe was an independent risk for disability in shopping and food preparation. A preventive strategy against WMH progression might lead to suppression of IADL disability and slow the progression to AD. Moreover, mood disturbance and mobility dysfunction were also predictive of IADL impairment. The prevalence of sarcopenia is increased in persons with aMCI and early AD [55]. Physical and psychological intervention should be provided in parallel from the early stage of AD.
